# Longitudinal Interaction Between Frailty and Postoperative Symptom Burden in Older Adults Undergoing Colorectal Cancer Surgery: A Random Intercept Cross-Lagged Panel Model Study

**DOI:** 10.3390/jcm15207954

**Published:** 2026-10-14

**Authors:** Pei Li, Lili Ge, Yang Liu, Keli Li, Tong Wu, Qingyu Fu, Yi Ding, Yun Liu, Qian Wu

**Affiliations:** 1 Department of Nursing, Shanghai Tenth People’s Hospital, School of Medicine, Tongji University, Shanghai 200072, China; lipe@tongji.edu.cn (P.L.); 2141365@tongji.edu.cn (L.G.); 2532523@tongji.edu.cn (K.L.); fanqieyu245@stu.ahtcm.edu.cn (Q.F.); 104754252258@henu.edu.cn (Y.D.); jiumangxing@jgsu.edu.cn (Y.L.); 2 Institute of Nursing and Health, School of Nursing and Health, Henan University, Kaifeng 475004, China; 104754241990@henu.edu.cn; 3 School of Medicine, Tongji University, Shanghai 200331, China; 2432752@tongji.edu.cn

**Keywords:** frailty, symptom burden, colorectal cancer, older adults, random intercept cross-lagged panel model, longitudinal study

## Abstract

**Background/Objectives**: Frailty and postoperative symptom burden are common in older adults undergoing colorectal cancer surgery, but their temporal associations remain unclear. This study examined longitudinal associations between frailty and two symptom dimensions, symptom severity and symptom interference, using the Random Intercept Cross-Lagged Panel Model (RI-CLPM). **Methods**: This prospective longitudinal study enrolled 242 older adults (≥60 years) undergoing CRC surgery. Frailty and symptoms were assessed before surgery and at 1 week, 1 month, 3 months, 6 months, and 12 months postoperatively. Separate RI-CLPMs were estimated for frailty with symptom severity and symptom interference. Sensitivity analyses compared results with conventional CLPMs, adjustment for time-varying albumin and body mass index, and a worst-case missing-data scenario. **Results**: Frailty and both symptom dimensions increased at 1 week after surgery, then generally declined. At the between-person level, frailty was strongly correlated with symptom severity and symptom interference. At the within-person level, higher symptom severity was associated with higher subsequent frailty from 1 to 3 months (*β* = 0.27, *p* = 0.033) and from 3 to 6 months (*β* = 0.38, *p* = 0.004), with the latter association remaining relatively stable across sensitivity analyses. Higher frailty was associated with higher subsequent symptom interference from before surgery to 1 week after surgery (*β* = 0.34), which was also relatively consistent across model specifications. Several other interval-specific paths changed after covariate adjustment or under the worst-case missing-data assumption. **Conclusions**: Frailty and postoperative symptom burden were closely associated at the between-person level, whereas within-person longitudinal associations were phase-specific and symptom-dimension-dependent.

## 1. Introduction

Colorectal cancer (CRC) is among the most common malignancies globally, with the majority of cases diagnosed in adults aged 60 years and older [1]. Surgical resection remains the primary curative strategy; however, older adults are particularly vulnerable to adverse postoperative outcomes [2]. Frailty, a multidimensional syndrome characterized by diminished physiological reserve and increased vulnerability to stressors, affects 20–50% of older adults undergoing CRC surgery and is associated with elevated morbidity and mortality and impaired health-related quality of life [3,4,5]. Postoperative symptom burden, encompassing pain, fatigue, sleep disturbance, and psychological distress, is equally prevalent and clinically consequential in this population, with persistent symptom burden documented up to one year following surgery [6,7,8,9]. In this context, symptom burden refers to a multidimensional construct capturing both symptom severity and symptom interference with daily functioning, as measured by composite patient-reported outcome instruments that aggregate across multiple domain [10,11]. This composite perspective is clinically relevant, as patients frequently experience simultaneous burden from multiple symptoms that collectively impair recovery and quality of life, beyond what any single symptom dimension might indicate [12,13].

The relationship between frailty and symptom burden is theoretically dynamically bidirectional. Frailty is associated with greater symptom severity through depleted physiological reserve and an attenuated stress response, while a high symptom burden whereas elevated symptom burden is associated with frailty progression by restricting physical activity, compromising nutritional status, and undermining rehabilitation engagement [14,15]. Longitudinal investigation of postoperative cancer rehabilitation demonstrated that symptom clusters are not isolated phenomena but are intimately associated with frailty status and quality of life, exhibiting dynamic evolution throughout the recovery process [16]. Further research on cognitive frailty showed significant associations between frailty and health-related quality of life, suggesting that frailty may exacerbate symptom perception and subsequently compromise functional outcomes [17]. Population-based large-scale analysis in the colorectal cancer context revealed that frailty severity substantially influenced time alive and at home following cancer surgery, with this effect partially mediated through postoperative complications and sustained symptom burden [18]. A study utilizing multi-state modeling further explored frailty trajectories among older cancer survivors, emphasizing the necessity of capturing such dynamic changes in longitudinal investigations [19]. These studies establish the theoretical foundation for a frailty-symptom framework.

However, these studies primarily relied on conventional cross-lagged panel models (CLPMs) or repeated-measures regression. Consequently, it remains unclear whether a transient increase in symptom burden within an individual prospectively predict frailty worsening, or whether the observed longitudinal associations merely reflect that patients with chronically high frailty also report persistently elevated symptoms. The Random Intercept Cross-Lagged Panel Model (RI-CLPM) addresses this limitation by partitioning variance into stable between-person components (random intercepts) and time-varying within-person deviations, thereby enabling less biased estimation of individual-level temporal dynamics and providing a more appropriate framework for examining the association between frailty and symptom burden [20,21].

Therefore, this study examined within-person bidirectional associations between frailty and postoperative symptom burden across six time points—preoperatively (T0) and at 1 week (T1), 1 month (T2), 3 months (T3), 6 months (T4), and 12 months (T5) postoperatively, using the RI-CLPM framework. We hypothesized that (1) frailty and symptom burden would show strong between-person correlations, reflecting shared stable determinants and (2) within-person bidirectional predictive effects would be observed such that each construct prospectively was associated with deviations in the other over time. The primary aim of this study was to examine bidirectional longitudinal associations between frailty and postoperative symptom burden across the surgical recovery trajectory, thereby providing observational evidence on their temporal sequencing that may inform future hypothesis-generating research.

## 2. Materials and Methods

### 2.1. Study Design and Setting

This was a single-center, prospective longitudinal study conducted at Shanghai Tenth People’s Hospital. Patient enrollment commenced on 27 February 2025 and concluded on 3 May 2025, spanning a 10-week enrollment window. Each enrolled participant then underwent prospective follow-up assessments at prespecified time points over 12 months postoperatively. The first participant completed the baseline assessment on 27 February 2025 and the final 12-month follow-up (T5) on 27 February 2026. The last participant was enrolled on 3 May 2025 and completed T5 on 3 May 2026. Thus, the overall study period extended from 27 February 2025 to 3 May 2026, ensuring that all participants completed the full six-wave assessment protocol (T0–T5) with 12 months of postoperative follow-up. A consolidated round of logic checks, range validation, and discrepancy resolution was performed from 4 to 13 May 2026, at which point the dataset was finalized and frozen for analysis. Written informed consent was obtained from all participants prior to enrollment.

### 2.2. Participants and Sample Size

Patients were considered eligible if they met the following inclusion criteria: (1) aged 60 years or older, (2) pathologically showed colorectal cancer, and (3) capable of communicating effectively and completing the questionnaires. The exclusion criteria were: (1) severe cognitive impairment or psychiatric disorders; (2) the presence of other active malignancies; (3) distant metastasis or multi-organ dysfunction; and (4) severe cardiac, hepatic, renal, or hematological diseases.

The target sample size was planned with reference to commonly used sample size heuristics for structural equation modeling and consideration of an anticipated attrition rate of approximately 20% over the 12-month follow-up period [22,23]. On this basis, a target enrollment of 260 participants was predefined. Given the complexity of the RI-CLPM, no formal simulation-based power analysis was performed. Accordingly, the planned sample size should be interpreted as a pragmatic design choice rather than evidence that the study was definitively powered for all model parameters, particularly the interval-specific within-person cross-lagged effects.

### 2.3. Data Collection and Procedures

Data were prospectively collected at six specific time points: preoperatively (T0) and at one week (T1), one month (T2), three months (T3), six months (T4), and 12 months (T5) postoperatively. Trained case managers administered standardized questionnaires using uniform instructions. Assessments were primarily conducted face-to-face during hospitalization or scheduled clinic visits; telephone interviews were utilized for participants who were unable to return to the hospital. To ensure data accuracy, all completed questionnaires were independently verified by two researchers prior to data entry.

### 2.4. Outcome Measures

#### 2.4.1. Frailty Assessment

Frailty was assessed using the Groningen Frailty Indicator (GFI), a widely validated instrument for older adults [24]. The GFI consists of 15 items evaluating frailty across physical, cognitive, social, and psychological domains [24]. Total scores range from 0 to 15, with higher scores indicating greater frailty severity [24]. In the current study, the Cronbach’s α coefficient for the Groningen Frailty Indicator (GFI) was 0.82.

#### 2.4.2. Postoperative Symptom Burden

The MD Anderson Symptom Inventory (MDASI) was utilized to measure postoperative symptom burden, which consists of two empirically validated dimensions: symptom severity and symptom interference [25]. The symptom severity subscale comprises 13 items assessing the intensity of core cancer-related symptoms, and the symptom interference subscale comprises 6 items assessing the degree to which symptoms interfere with daily functioning. Each item is rated on a numeric scale from 0 to 10. For each subscale, a mean score ranging from 0 to 10 was calculated by averaging the corresponding items, with higher scores indicating greater symptom severity or interference. This dimensional scoring approach aligns with the original MDASI validation framework and represents the conventional scoring method recommended for the instrument [25]. The Chinese version of the MDASI has been well-validated in Chinese cancer populations [26]. In the current study, the Cronbach’s α coefficients for the MD Anderson Symptom Inventory (MDASI) were 0.86 for the symptom severity subscale and 0.84 for the symptom interference subscale.

### 2.5. Covariates

Sociodemographic variables included age, sex, educational level, income, occupation, marital status, and Body Mass Index (BMI). Clinical variables comprised comorbidities, tumor location, histological type, TNM stage, surgical approach, chemotherapy regimen and cycles, relevant laboratory indices, and the Nutritional Risk Screening 2002 (NRS 2002) score.

### 2.6. Statistical Analysis

#### 2.6.1. Model Specification and Estimation

Descriptive statistics and baseline comparisons were performed using R version 4.4.2. The RI-CLPMs were fitted using the lavaan package (version 0.6-19) in R software [20]. In this model, each observed variable at each time point was decomposed into a time-invariant between-person component (random intercept) and a time-varying within-person component [20]. Separate RI-CLPMs were fitted for the two MDASI dimensions. For each model, two random intercepts were specified: one for frailty (GFI total score) and one for the respective MDASI subscale (symptom severity or symptom interference). Factor loadings were constrained to unity across all six waves to ensure model identification [27].

At the within-person level, autoregressive paths (reflecting the carry-over of individual deviations from one’s own expected mean) and cross-lagged paths (reflecting prospective within-person spillover effects between the two constructs) were freely estimated for each consecutive time interval (T0 → T1, T1 → T2, T2 → T3, T3 → T4, and T4 → T5). This unconstrained approach was selected given the unequal time spacing between measurement occasions. Within-time residual covariances between frailty and the respective MDASI subscale were also estimated at each wave to account for concurrent associations not explained by the lagged effects. Crucially, because the RI-CLPM inherently controls for all time-invariant unobserved and observed inter-individual differences via the random intercepts [20], the aforementioned sociodemographic and clinical covariates were solely utilized for baseline descriptive statistics and to assess potential attrition bias, thereby preserving model parsimony. Model parameters were estimated using Maximum Likelihood with Robust standard errors (MLR) to accommodate potential non-normality in the data.

Missing data were handled via Full Information Maximum Likelihood (FIML) estimation under the assumption of Missing at Random (MAR), which yields unbiased parameter estimates when missingness is related to observed variables [28,29]. Baseline sociodemographic and clinical characteristics were compared between participants with complete follow-up and those with any missing data to assess the plausibility of the MAR assumption, with independent-samples *t*-tests applied for continuous variables and χ^2^ tests for categorical variables. Multivariable logistic regression was further conducted to quantify the explanatory power of baseline characteristics for missingness. MAR represents an untestable working assumption, and baseline comparisons cannot rule out MNAR mechanisms driven by unmeasured outcomes such as disease progression or mortality.

#### 2.6.2. Model Fit Evaluation

Model fit was evaluated using the Comparative Fit Index (CFI), Tucker–Lewis Index (TLI), Root Mean Square Error of Approximation (RMSEA) with its 95% confidence interval (CI), and Standardized Root Mean Square Residual (SRMR). A good fit was defined as CFI and TLI ≥ 0.95, RMSEA ≤ 0.06, and SRMR ≤ 0.08; an adequate fit was defined as CFI and TLI ≥ 0.90, RMSEA ≤ 0.08, and SRMR ≤ 0.08 [29].

#### 2.6.3. Sensitivity Analyses

To evaluate the robustness of the primary findings, two sets of sensitivity analyses were performed for each of the two MDASI subscale models.

First, to assess the impact of partitioning between-person variance on cross-lagged estimates, conventional cross-lagged panel models (CLPMs) without random intercepts were fitted for each MDASI subscale; their fit indices and standardized path coefficients were compared with those of the corresponding primary RI-CLPMs.

Second, because nutritional status may confound the relationship between symptom burden and frailty, adjusted RI-CLPMs were fitted for each MDASI subscale, incorporating time-varying serum albumin and body mass index (BMI) as covariates at each postoperative assessment (T1–T5). These nutritional indicators were selected a priori based on their established associations with both postoperative symptomatology and geriatric frailty. Path coefficients from the adjusted models were compared with those from the primary unadjusted models to assess the stability of the cross-lagged effects.

Third, a worst-case scenario sensitivity analysis was performed to examine the potential impact of missing not at random (MNAR) attributable to disease progression or mortality. For participants with permanent dropout, frailty and MDASI subscale scores at all subsequent time points were imputed to the 90th percentile of the observed score distribution at each corresponding wave. RI-CLPMs were subsequently refitted with these imputed values, and cross-lagged path coefficients were compared with those from the primary MAR-based analyses to evaluate the stability of findings under a plausible MNAR scenario.

For all sensitivity analyses, a two-tailed *p*-value < 0.05 was considered statistically significant.

## 3. Results

### 3.1. Participant Characteristics

**Figure 1:**
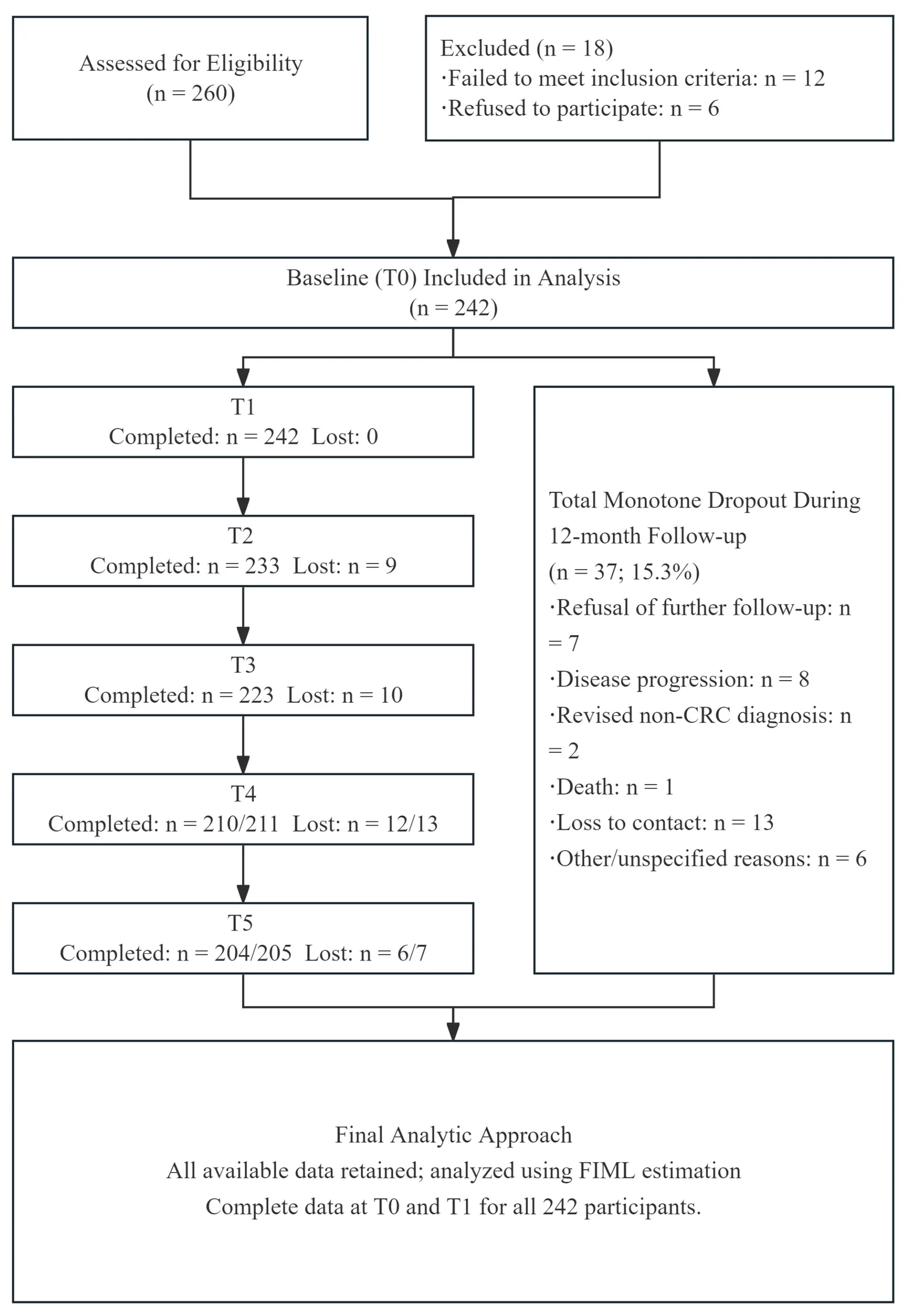
Participant flow diagram.

Baseline enrollment screened 260 patients, of whom 18 were excluded prior to baseline assessment (T0) due to failure to meet inclusion criteria (*n* = 12) or refusal to participate (*n* = 6). The final analytic sample comprised 242 participants at baseline; the baseline characteristics of these participants are detailed in Table 1. Among all 242 participants, 39 (16.1%) had at least one missing assessment during the 12-month follow-up. Missingness was concentrated at later time points and followed a predominantly monotone trajectory, with 9 (3.7%), 10 (4.1%), 12 (5.0%), and 6 (2.0%) new dropouts occurring between T1–T2, T2–T3, T3–T4, and T4–T5, respectively. At the final 12-month assessment (T5), 37 participants (15.3%) had missing GFI data and 38 (15.7%) had missing MDASI data. Reasons for dropout included refusal of further follow-up (*n* = 7), disease progression (*n* = 8), revised non-CRC diagnosis (*n* = 2), death during follow-up (*n* = 1), and loss to contact (*n* = 13). Only 3 participants (1.2%) exhibited intermittent missingness at a single time point, with complete data available at all other assessments. All data were retained and analyzed using FIML estimation. Complete data were available for all 242 participants at T0 and T1; subsequently, missingness increased monotonically, with 37 (15.3%) and 38 (15.7%) participants missing GFI and MDASI data, respectively, at T5. The participant flow throughout the six assessment waves is shown in Figure 1. Eight distinct missing-data patterns were identified, with monotone dropout (permanent attrition) accounting for over 90% of all missing cases, and non-monotone (intermittent) missingness being extremely rare (<2%). The full distribution of missing data is presented in Table 2, and detailed missing-data patterns are presented in Table 3.

### 3.2. Descriptive Statistics of Frailty and Symptom Burden Across Time Points

Descriptive statistics for GFI and the two MDASI dimensions (symptom severity and interference) across the six time points are presented in Table 4. Both frailty and mean symptom interference scores peaked at T1 (GFI = 4.60 ± 2.20; MDASI interference = 3.20 ± 1.78). Frailty subsequently declined with some fluctuation, whereas symptom interference decreased steadily through T5. Mean symptom severity scores remained relatively stable from T1 to T4 (1.40–1.44), with a slight peak at T3 (1.44 ± 1.18). The lowest scores for all three measures were observed at T5 (GFI = 2.53 ± 2.72; MDASI severity = 0.89 ± 1.06; MDASI interference = 1.15 ± 1.82).

**Figure 2:**
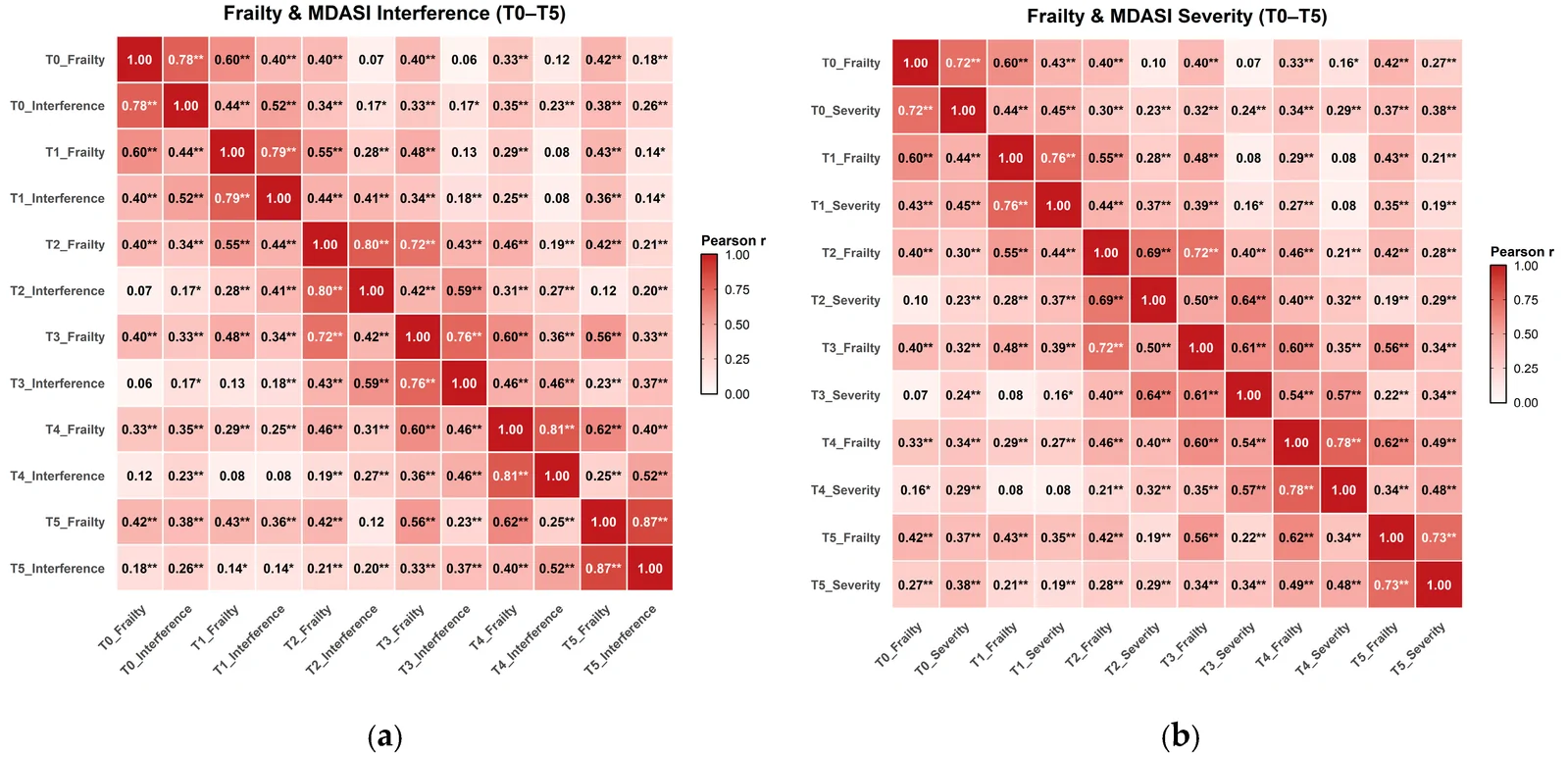
Heat maps of the associations between frailty and (**a**) symptom interference and (**b**) symptom severity across different time points. * *p* < 0.05, ** *p* < 0.001.

As illustrated in Figure 2 and the two correlation heatmaps, strong cross-sectional associations were observed between frailty and both symptom severity (*r* = 0.61–0.78) and symptom interference (*r* = 0.76–0.87) at each time point, with consistently stronger associations for symptom interference. Across adjacent time intervals, cross-construct correlations were positive and weak to moderate, ranging from *r* = 0.28 to 0.54 for frailty and symptom severity and from *r* = 0.25 to 0.46 for frailty and symptom interference. Moderate to strong temporal stability across adjacent assessments was also evident for frailty (*r* = 0.55–0.72), symptom severity (*r* = 0.37–0.64), and symptom interference (*r* = 0.41–0.59). These patterns provided a basis for further examining the longitudinal relationships between frailty and each symptom dimension using cross-lagged panel modeling.

### 3.3. RI-CLPM Results

#### 3.3.1. Model 1: Frailty and MDASI Symptom Severity

The RI-CLPM results for frailty and MDASI symptom severity are shown in Table 5. In Model 1, the between-person random intercept for GFI was positively correlated with the between-person random intercept for MDASI symptom severity (*r* = 0.747, 95% CI [0.686, 0.798], *p* < 0.001).

At the within-person level, the autoregressive paths for GFI varied across adjacent intervals. From T0 to T1, the autoregressive path was positive but not statistically significant (B = 0.037, SE = 0.077, *β* = 0.045, 95% CI [−0.138, 0.228], *p* = 0.629). From T1 to T2, the estimate was negative and not statistically significant (B = −0.048, SE = 0.091, *β* = −0.046, 95% CI [−0.218, 0.126], *p* = 0.600). From T2 to T3, the estimate was again positive and not statistically significant (B = 0.075, SE = 0.073, *β* = 0.092, 95% CI [−0.086, 0.270], *p* = 0.309). From T3 to T4, the autoregressive path for GFI was negative and statistically significant (B = −0.532, SE = 0.135, *β* = −0.346, 95% CI [−0.520, −0.172], *p* < 0.001). From T4 to T5, the estimate was positive and did not reach statistical significance (B = 0.166, SE = 0.097, *β* = 0.177, 95% CI [−0.028, 0.381], *p* = 0.090).

The autoregressive paths for MDASI symptom severity also showed variation across time. From T0 to T1, the autoregressive path was positive and statistically significant (B = 0.239, SE = 0.097, *β* = 0.227, 95% CI [0.045, 0.409], *p* = 0.015). From T1 to T2, the estimate was negative and not statistically significant (B = −0.103, SE = 0.083, *β* = −0.108, 95% CI [−0.279, 0.064], *p* = 0.216). From T2 to T3, the autoregressive coefficient was positive and statistically significant (B = 0.200, SE = 0.090, *β* = 0.203, 95% CI [0.023, 0.382], *p* = 0.027). From T3 to T4, a positive and statistically significant autoregressive path was again observed (B = 0.285, SE = 0.125, *β* = 0.211, 95% CI [0.028, 0.394], *p* = 0.024). From T4 to T5, the estimate was close to zero and not statistically significant (B = −0.000, SE = 0.082, *β* = −0.000, 95% CI [−0.206, 0.206], *p* = 0.999).

For the cross-lagged paths from GFI to MDASI symptom severity, the estimates from T0 to T1, T1 to T2, and T2 to T3 were all small and not statistically significant, with coefficients of B = −0.009, SE = 0.043, *β* = −0.020, 95% CI [−0.203, 0.162], *p* = 0.826; B = 0.012, SE = 0.046, *β* = 0.023, 95% CI [−0.148, 0.195], *p* = 0.789; and B = 0.022, SE = 0.046, *β* = 0.044, 95% CI [−0.135, 0.223], *p* = 0.631, respectively. From T3 to T4, the cross-lagged path from GFI to subsequent symptom severity was negative and statistically significant (B = −0.199, SE = 0.080, *β* = −0.231, 95% CI [−0.414, −0.049], *p* = 0.013). From T4 to T5, the estimate remained negative but was not statistically significant (B = −0.041, SE = 0.045, *β* = −0.095, 95% CI [−0.301, 0.111], *p* = 0.363).

For the cross-lagged paths from MDASI symptom severity to GFI, the estimate from T0 to T1 was positive and approached statistical significance (B = 0.309, SE = 0.174, *β* = 0.165, 95% CI [−0.018, 0.348], *p* = 0.077). From T1 to T2, the estimate was negative and not statistically significant (B = −0.057, SE = 0.163, *β* = −0.030, 95% CI [−0.202, 0.142], *p* = 0.729). From T2 to T3, a positive and statistically significant cross-lagged path was observed (B = 0.308, SE = 0.142, *β* = 0.195, 95% CI [0.018, 0.373], *p* = 0.031). From T3 to T4, the positive cross-lagged coefficient increased further and remained statistically significant (B = 1.061, SE = 0.213, *β* = 0.439, 95% CI [0.265, 0.613], *p* < 0.001). From T4 to T5, the estimate was negative and not statistically significant (B = −0.211, SE = 0.176, *β* = −0.124, 95% CI [−0.329, 0.080], *p* = 0.233).

Across the within-person estimates in Model 1, statistically significant positive coefficients were observed for the autoregressive paths of symptom severity from T0 to T1, from T2 to T3, and from T3 to T4, as well as for the cross-lagged paths from symptom severity to GFI from T2 to T3 and from T3 to T4. Statistically significant negative coefficients were observed for the GFI autoregressive path from T3 to T4 and for the cross-lagged path from GFI to symptom severity over the same interval.

#### 3.3.2. Model 2: Frailty and MDASI Symptom Interference

In Model 2, the between-person random intercept for GFI was positively correlated with the between-person random intercept for MDASI symptom interference (*r* = 0.865, 95% CI [0.830, 0.894], *p* < 0.001).

At the within-person level, the autoregressive paths for GFI showed both positive and negative estimates across intervals. From T0 to T1, the autoregressive path was positive and statistically significant (B = 0.272, SE = 0.075, *β* = 0.328, 95% CI [0.150, 0.506], *p* < 0.001). From T1 to T2, the estimate was negative and not statistically significant (B = −0.143, SE = 0.097, *β* = −0.138, 95% CI [−0.322, 0.046], *p* = 0.141). From T2 to T3, the autoregressive path was positive and statistically significant (B = 0.151, SE = 0.074, *β* = 0.186, 95% CI [0.007, 0.365], *p* = 0.042). From T3 to T4, the estimate was negative and not statistically significant (B = −0.161, SE = 0.142, *β* = −0.105, 95% CI [−0.287, 0.078], *p* = 0.259). From T4 to T5, the estimate was positive and not statistically significant (B = 0.144, SE = 0.099, *β* = 0.153, 95% CI [−0.055, 0.361], *p* = 0.148).

The autoregressive paths for MDASI symptom interference were not statistically significant at any interval. The estimates were B = −0.123, SE = 0.085, *β* = −0.131, 95% CI [−0.310, 0.048], *p* = 0.150 from T0 to T1; B = −0.041, SE = 0.089, *β* = −0.043, 95% CI [−0.227, 0.141], *p* = 0.645 from T1 to T2; B = 0.051, SE = 0.083, *β* = 0.056, 95% CI [−0.123, 0.236], *p* = 0.537 from T2 to T3; B = −0.158, SE = 0.129, *β* = −0.114, 95% CI [−0.297, 0.069], *p* = 0.222 from T3 to T4; and B = 0.115, SE = 0.097, *β* = 0.125, 95% CI [−0.083, 0.333], *p* = 0.238 from T4 to T5.

For the cross-lagged paths from GFI to MDASI symptom interference, a positive and statistically significant association was observed from T0 to T1 (B = 0.197, SE = 0.063, *β* = 0.284, 95% CI [0.105, 0.462], *p* = 0.002). The estimate from T1 to T2 was positive but not statistically significant (B = 0.072, SE = 0.074, *β* = 0.091, 95% CI [−0.093, 0.274], *p* = 0.333). From T2 to T3, the coefficient remained positive and was close to statistical significance (B = 0.121, SE = 0.062, *β* = 0.177, 95% CI [−0.003, 0.356], *p* = 0.054). The estimates from T3 to T4 and from T4 to T5 were small and not statistically significant (B = 0.009, SE = 0.111, *β* = 0.008, 95% CI [−0.176, 0.191], *p* = 0.934; B = −0.008, SE = 0.075, *β* = −0.012, 95% CI [−0.220, 0.196], *p* = 0.910, respectively).

For the cross-lagged paths from MDASI symptom interference to GFI, the estimate from T0 to T1 was negative and statistically significant (B = −0.255, SE = 0.102, *β* = −0.227, 95% CI [−0.405, −0.049], *p* = 0.013). The estimates for the subsequent intervals were not statistically significant, including B = 0.127, SE = 0.117, *β* = 0.101, 95% CI [−0.083, 0.286], *p* = 0.279 from T1 to T2, B = 0.064, SE = 0.098, *β* = 0.059, 95% CI [−0.120, 0.238], *p* = 0.516 from T2 to T3, B = 0.140, SE = 0.165, *β* = 0.079, 95% CI [−0.104, 0.261], *p* = 0.396 from T3 to T4, and B = −0.112, SE = 0.129, *β* = −0.091, 95% CI [−0.300, 0.117], *p* = 0.388 from T4 to T5.

The RI-CLPM results for frailty and MDASI symptom interference are shown in Table 6.Within Model 2, statistically significant positive coefficients were observed for the GFI autoregressive paths from T0 to T1 and from T2 to T3, as well as for the cross-lagged path from GFI to symptom interference from T0 to T1. A statistically significant negative coefficient was observed for the cross-lagged path from symptom interference to GFI from T0 to T1.

### 3.4. Sensitivity Analysis Results

#### 3.4.1. RI-CLPM vs. Conventional CLPM

For the model linking GFI and MDASI symptom severity, the conventional CLPM showed an overall adequate fit (CFI = 0.918, TLI = 0.901, RMSEA = 0.071, 90% CI [0.063, 0.079], SRMR = 0.067), whereas the corresponding RI-CLPM in the main analysis showed a slightly better fit (CFI = 0.931, TLI = 0.914, RMSEA = 0.064, 90% CI [0.056, 0.072], SRMR = 0.061). Although both models met the criteria for adequate fit, the RI-CLPM showed modest improvement in global fit and yielded a different pattern of interval-specific cross-lagged estimates.

Specifically, in the RI-CLPM, statistically significant cross-lagged effects were observed from MDASI symptom severity to GFI at T2 → T3 (*β* = 0.195, *p* = 0.031) and T3 → T4 (*β* = 0.439, *p* < 0.001) and from GFI to MDASI symptom severity at T3 → T4 (*β* = −0.231, *p* = 0.013). By contrast, in the conventional CLPM, statistically significant cross-lagged paths were observed from GFI to symptom severity at T0 → T1 (*β* = 0.216, *p* = 0.009) and T4 → T5 (*β* = 0.297, *p* = 0.002), and from symptom severity to GFI at T3 → T4 (*β* = 0.251, *p* < 0.001) and T4 → T5 (*β* = −0.354, *p* < 0.001). Thus, the timing, direction, and significance of several cross-lagged coefficients differed between the two modeling approaches.

For the model linking GFI and MDASI symptom interference, the conventional CLPM also showed adequate fit (CFI = 0.924, TLI = 0.907, RMSEA = 0.069, 90% CI [0.061, 0.077], SRMR = 0.064), while the RI-CLPM showed slightly better fit (CFI = 0.938, TLI = 0.921, RMSEA = 0.061, 90% CI [0.053, 0.069], SRMR = 0.058). In the RI-CLPM, significant cross-lagged effects were limited to T0 → T1 in both directions, with a positive path from GFI to symptom interference (*β* = 0.284, *p* = 0.002) and a negative path from symptom interference to GFI (*β* = −0.227, *p* = 0.013). In contrast, the conventional CLPM identified a larger number of significant GFI-to-interference paths, including T0 → T1 (*β* = 0.208, *p* = 0.021), T1 → T2 (*β* = 0.184, *p* = 0.036), T3 → T4 (*β* = 0.261, *p* = 0.005), and T4 → T5 (*β* = 0.272, *p* = 0.005), whereas none of the interference-to-GFI paths reached statistical significance.

Taken together, comparison with the conventional CLPM showed that the estimated cross-lagged associations were sensitive to whether stable between-person differences were explicitly separated from within-person fluctuations.

#### 3.4.2. Robustness to Time-Varying Nutritional Covariates

After adjustment for time-varying serum albumin and body mass index at T1–T5, the RI-CLPM for MDASI symptom severity showed adequate fit (CFI = 0.919, TLI = 0.902, RMSEA = 0.068, 90% CI [0.060, 0.076], SRMR = 0.066). Compared with the unadjusted RI-CLPM, the overall fit was slightly attenuated, which was consistent with the increased model complexity after inclusion of repeated covariates. Nevertheless, the overall pattern of the cross-lagged estimates remained partially similar.

In the adjusted severity model, a statistically significant positive cross-lagged path from MDASI symptom severity to GFI remained at T3 → T4 (*β* = 0.208, *p* = 0.003). In contrast, the path from symptom severity to GFI at T2 → T3 was no longer statistically significant. For the opposite direction, the adjusted model showed a statistically significant positive path from GFI to symptom severity at T0 → T1 (*β* = 0.202, *p* = 0.014) and at T4 → T5 (*β* = 0.275, *p* = 0.001), whereas the negative GFI-to-severity path at T3 → T4 observed in the main RI-CLPM was attenuated and no longer statistically significant (*β* = −0.003, *p* = 0.966). Overall, adjustment for albumin and body mass index altered the significance pattern of several interval-specific cross-lagged coefficients, although some directional associations remained.

For the MDASI symptom interference model, the nutritionally adjusted RI-CLPM also showed adequate fit (CFI = 0.926, TLI = 0.909, RMSEA = 0.066, 90% CI [0.058, 0.074], SRMR = 0.063). In the main RI-CLPM, significant cross-lagged effects had been confined to T0 → T1 in both directions. After adjustment, the positive GFI-to-interference path at T0 → T1 remained statistically significant (*β* = 0.208, *p* = 0.020), while additional positive GFI-to-interference paths emerged at T3 → T4 (*β* = 0.238, *p* = 0.009) and T4 → T5 (*β* = 0.215, *p* = 0.020). By contrast, the negative interference-to-GFI path at T0 → T1 observed in the unadjusted RI-CLPM was no longer statistically significant after adjustment (*β* = −0.012, *p* = 0.886), and none of the interference-to-GFI paths reached statistical significance in the adjusted model.

Overall, inclusion of time-varying nutritional covariates resulted in some interval-specific changes in statistical significance, but several cross-lagged paths remained directionally consistent with the main RI-CLPM.

#### 3.4.3. Worst-Case Scenario Sensitivity Analysis for MNAR

Under the worst-case MNAR scenario, the RI-CLPM for MDASI symptom severity showed adequate, though slightly weaker, fit than the main RI-CLPM (CFI = 0.912, TLI = 0.895, RMSEA = 0.074, 90% CI [0.066, 0.082], SRMR = 0.070). This pattern was consistent with the fact that deterministic assignment of high post-dropout values increased distributional asymmetry and reduced model-data correspondence relative to the missing-at-random primary analysis.

Within this worst-case severity model, the cross-lagged path from symptom severity to GFI at T3 → T4 remained positive and statistically significant (*β* = 0.275, *p* < 0.001). In addition, the path from symptom severity to GFI at T4 → T5 became negative and statistically significant (*β* = −0.225, *p* = 0.005). For the opposite direction, the path from GFI to symptom severity at T0 → T1 was positive and statistically significant (*β* = 0.216, *p* = 0.009), and the T4 → T5 path also became positive and statistically significant (*β* = 0.334, *p* < 0.001), whereas the T3 → T4 path was attenuated and no longer statistically significant (*β* = 0.125, *p* = 0.068). Compared with the main RI-CLPM, the interval-specific significance pattern therefore changed under the worst-case MNAR assumption.

For the MDASI symptom interference model, the worst-case MNAR RI-CLPM likewise showed adequate fit (CFI = 0.918, TLI = 0.901, RMSEA = 0.071, 90% CI [0.063, 0.079], SRMR = 0.068). In this model, the cross-lagged path from GFI to symptom interference remained positive and statistically significant at T0 → T1 (*β* = 0.208, *p* = 0.021) and additionally reached significance at T3 → T4 (*β* = 0.303, *p* < 0.001) and T4 → T5 (*β* = 0.317, *p* < 0.001). The symptom interference-to-GFI path at T0 → T1 was no longer statistically significant (*β* = 0.002, *p* = 0.979), whereas the T2 → T3 path became positive and statistically significant (*β* = 0.187, *p* = 0.015). Thus, under the worst-case MNAR scenario, the interference model also showed changes in the location and direction of statistically significant cross-lagged effects.

Overall, the worst-case MNAR analyses suggested that some cross-lagged findings persisted, whereas other interval-specific associations changed in significance or direction, indicating that selected estimates were sensitive to assumptions about the missing-data mechanism.

## 4. Discussion

### 4.1. Stable Between-Person Co-Occurrence Was More Consistent than Interval-Specific Within-Person Dynamics

A first notable finding was that, across both MDASI subscale models, the between-person associations were consistently positive and substantial. Participants with higher average frailty across follow-up also tended to report higher average symptom burden, whether symptom burden was indexed by symptom severity or symptom interference. This pattern was more stable than the within-person cross-lagged estimates and suggests that the co-occurrence between frailty and symptom burden may be more robust at the level of enduring individual differences than at the level of short-term deviations from a person’s expected trajectory.

This distinction is important because prior observational research has often reported that frailty and symptom burden are closely linked, but many studies have relied on cross-sectional or conventional longitudinal approaches that do not clearly separate between-person from within-person sources of association. The present findings suggest that a considerable portion of the frailty–symptom relationship may reflect relatively stable patient-level differences that persist across follow-up, rather than a uniform interval-to-interval reciprocal pattern. In that sense, the results are broadly consistent with existing literature showing clustering of frailty, functional vulnerability, and symptom burden in older adults while also refining that literature by showing that strong cross-sectional or person-level associations do not necessarily translate into consistent within-person cross-lagged effects at every time interval.

### 4.2. The Concentration of Severity-Related Associations in the Middle Follow-Up Intervals May Reflect Delayed Consolidation of Postoperative Vulnerability

In the symptom severity model, the more reproducible within-person signal appeared in the middle portion of follow-up, particularly around T3 → T4, where symptom severity was positively associated with subsequent frailty in the main RI-CLPM and remained evident across sensitivity analyses. This interval-specific concentration is unlikely to be random noise alone, because it was not confined to a single isolated coefficient and because the T3 → T4 severity-to-frailty path was one of the few associations that retained significance across more than one analytical specification.

A plausible interpretation is that the symptom severity dimension may become more strongly aligned with frailty after the immediate postoperative phase, when early acute fluctuations begin to settle and the cumulative burden of persistent symptoms becomes more apparent. In many surgical oncology trajectories, the earliest period after surgery is characterized by short-term instability, active symptom management, and rapid changes in recovery status. Under such circumstances, momentary symptom severity may reflect a mixture of transient postoperative effects and individual recovery variation, making its association with later frailty less consistent. By contrast, when symptom severity remains elevated into the later recovery phase, it may better track sustained physiological vulnerability, reduced reserve, or broader recovery difficulties that are also captured by frailty measures. Within this framework, the T3 → T4 interval may represent a period in which symptom burden is less dominated by immediate postoperative noise and more reflective of an ongoing vulnerable state.

This interpretation is also compatible with prior longitudinal literature showing that frailty in older adults is often more strongly associated with persistent or accumulated health deficits than with isolated short-term symptoms. It also aligns with symptom science research suggesting that chronicity and persistence of symptoms may be more prognostically informative than transient peaks. Thus, the present findings do not suggest that symptom severity is uniformly associated with later frailty across all postoperative periods; rather, they suggest that the association may become more detectable once postoperative recovery enters a more consolidated phase.

### 4.3. The Early Prominence of Interference-Related Associations May Reflect the Salience of Functional Disruption Immediately After Surgery

In contrast to symptom severity, symptom interference showed its clearest within-person associations in the earliest interval, T0 → T1. In the main RI-CLPM, frailty was positively associated with later interference, and interference was negatively associated with later frailty, both within this early interval. Across sensitivity analyses, the frailty-to-interference association at T0 → T1 was among the more recurrent findings, even when other interference-related paths changed under alternative specifications.

One possible explanation is that symptom interference may be especially sensitive to the immediate functional consequences of surgery and early recovery demands. Unlike symptom severity, which captures intensity of symptom experience, symptom interference reflects the extent to which symptoms disrupt daily functioning, activity, mood, or role performance. In the early postoperative period, these domains may be especially labile because patients are adapting to surgery-related limitations, temporary reductions in mobility or self-care, and abrupt changes in routine. Within such a context, baseline frailty may be more closely aligned with how strongly postoperative symptoms translate into day-to-day disruption, making early frailty-to-interference associations more detectable.

This temporal pattern may also help explain why interference-related associations were less consistent later in follow-up. As recovery progresses, patients may adapt functionally even when some symptoms persist, which could weaken the correspondence between symptom burden and interference over longer intervals. In other words, interference may be particularly informative during the early adjustment period, whereas later in follow-up, it may be shaped not only by symptom levels but also by adaptation, support, coping, rehabilitation, and changes in role expectations. This interpretation is consistent with prior symptom research showing that symptom severity and symptom interference, although correlated, often follow partially distinct trajectories and should not be treated as interchangeable indicators.

The negative interference-to-frailty path at T0 → T1 in the main RI-CLPM should be interpreted cautiously. Because this finding did not persist after covariate adjustment and disappeared under the worst-case MNAR scenario, it appears less stable than the frailty-to-interference path in the same interval. Therefore, among the interference-related findings, the more defensible takeaway is not a robust bidirectional pattern, but rather a relatively more consistent early association from frailty to subsequent symptom interference.

### 4.4. Differences Between Symptom Severity and Symptom Interference Suggest That Symptom Burden Is Multidimensional Rather than Temporally Uniform

Taken together, the two MDASI subscale models suggest that symptom burden was not longitudinally related to frailty in a single uniform way. Symptom severity showed its clearest associations in the middle recovery intervals, whereas symptom interference showed its clearest association in the earliest interval. This contrast is meaningful because it indicates that the temporal relationship with frailty depends on which aspect of symptom burden is being modeled.

This finding adds nuance to the existing literature. Studies that collapse symptom burden into a single global construct may obscure important differences between symptom intensity and symptom-related disruption. Severity may be more closely aligned with sustained physiological or clinical burden once the acute recovery stage has passed, whereas interference may be more sensitive to immediate disruptions in functioning during early postoperative adaptation. Thus, the present results support the view that symptom burden is not only multidimensional in content but also multidimensional in temporal patterning.

From a theoretical perspective, these findings are more consistent with a staged or phase-dependent association structure than with a single stable reciprocal model spanning the full follow-up period. They suggest that frailty may be linked to different dimensions of symptom experience at different stages of recovery. This does not establish a directional mechanism, but it does challenge any assumption that one summary symptom measure would fully capture the relevant longitudinal relationship with frailty across all intervals.

### 4.5. Differences Between RI-CLPM and Conventional CLPM Indicate That Some Apparent Reciprocal Associations May Reflect Stable Between-Person Heterogeneity

One of the most important findings from the sensitivity analyses was that the pattern of cross-lagged estimates changed substantially when the conventional CLPM was used instead of the RI-CLPM. In both the severity and interference models, the conventional CLPM yielded more numerous or differently timed significant paths, especially for frailty-to-symptom associations. This suggests that some associations that appear prominent in a conventional CLPM may be influenced by stable between-person differences rather than reflecting pure within-person temporal coupling.

This issue is not merely technical; it has interpretive consequences. Conventional CLPMs are widely used in longitudinal health research, but they conflate stable person-level differences with within-person changes over time. When frailty and symptom burden are both strongly patterned by enduring characteristics such as baseline health status, resilience, multimorbidity, or recovery capacity, conventional CLPM coefficients may partially capture those stable differences. The RI-CLPM, by partitioning out random intercept variance, provides a more restrictive test of whether deviations from a person’s own expected level are associated with subsequent deviations in the other construct.

The fact that some significant CLPM paths disappeared or changed direction in the RI-CLPM therefore suggests that at least part of the apparent reciprocity in simpler models may arise from between-person heterogeneity. This interpretation is consistent with methodological work showing that CLPM and RI-CLPM can produce substantially different conclusions when stable trait-like differences are present. Accordingly, the current findings extend that methodological point into the context of frailty and postoperative symptom burden in older adults. For teaching purposes, this may be one of the clearest messages from the study: the substantive conclusion about “who predicts whom” depends materially on whether the model distinguishes stable differences between individuals from within-person fluctuations over time.

### 4.6. Changes After Adjustment for Albumin and Body Mass Index Suggest Partial Overlap Between Nutritional Status and Frailty–Symptom Associations

Adjustment for time-varying albumin and body mass index altered several interval-specific cross-lagged coefficients in both models. Some paths that were significant in the unadjusted RI-CLPM were attenuated, whereas other paths became statistically significant after adjustment. This pattern may seem counterintuitive at first, but from a statistical and clinical perspective, it is plausible.

One reason is that nutritional indicators may share variance with both frailty and symptom burden, but not necessarily in the same way at every interval or for every symptom dimension. When a time-varying covariate is associated with both constructs, adjustment can reduce an observed cross-lagged coefficient if that coefficient partly reflects variance common to nutritional status. This may explain why some originally significant paths, such as the early interference-to-frailty path or the T2 → T3 severity-to-frailty path, were no longer statistically significant after adjustment. In these cases, part of the original association may have overlapped with time-varying nutritional status.

At the same time, adjustment can also make some paths more visible. If albumin or body mass index introduce heterogeneity or suppressor-like distortion in the unadjusted model, controlling for them can clarify the remaining association between frailty and symptom burden. This may help explain why certain frailty-to-symptom paths emerged after adjustment, including the T0 → T1 and T4 → T5 frailty-to-severity paths and the later frailty-to-interference paths. In observational longitudinal settings, such changes do not necessarily imply contradiction; rather, they indicate that the association structure is partly contingent on shared variance with nutritional status.

These findings fit with prior literature showing that nutritional status is closely related to both symptom burden and frailty in older surgical populations. The present results add a longitudinal modeling perspective by suggesting that nutritional status may not simply act as a background characteristic but may reshape how strongly frailty and symptoms are statistically linked at different recovery stages. At the same time, because some associations remained directionally similar after adjustment, the data do not support the view that nutritional covariates fully account for all observed frailty–symptom associations.

### 4.7. Sensitivity to the Worst-Case MNAR Scenario Suggests That Later Interval Estimates Are More Vulnerable to Dropout Assumptions

The worst-case MNAR sensitivity analysis showed that several interval-specific paths changed in significance or direction when permanently dropped-out participants were assigned high post-dropout frailty and symptom values. This finding is methodologically informative. In longitudinal postoperative studies with older adults, dropout related to disease progression, clinical deterioration, or death is unlikely to be completely benign, and later follow-up intervals are especially vulnerable to this problem. When missingness is associated with poorer underlying status, analyses based only on observed data under missing-at-random assumptions may underrepresent worse trajectories.

The changes seen under the MNAR scenario were particularly notable in later intervals, where additional significant frailty-to-symptom paths emerged and some earlier findings weakened. This pattern is consistent with the possibility that attrition disproportionately removed participants with higher frailty or greater symptom burden from the observed later-wave data. Once those missing trajectories were pushed toward worse values, the modeled associations at later intervals became stronger or changed location. This does not establish what the true missing-data process was, but it indicates that some later cross-lagged estimates were less robust to alternative assumptions about dropout.

Importantly, not all findings were equally affected. In the severity model, the T3 → T4 symptom severity-to-frailty path remained statistically significant even under the worst-case scenario, which strengthens its standing as one of the relatively more stable interval-specific associations in the study. By contrast, several other paths appeared more assumption-sensitive. Thus, the MNAR analysis helps differentiate between findings that were comparatively persistent across specifications and those that depended more heavily on how post-dropout outcomes were handled.

### 4.8. The Most Defensible Substantive Conclusion Is the Presence of Selected, Phase-Specific Associations Rather than a Single Stable Bidirectional Pattern

Taken together, the main analyses and sensitivity analyses do not support a simple claim of a uniform bidirectional relationship between frailty and symptom burden across the entire follow-up period. Instead, the evidence points to selected, phase-specific associations whose detectability depends partly on how symptom burden is defined and how the longitudinal model is specified.

Within that broader caution, two findings appear relatively more defensible than others. First, the association between higher symptom severity and subsequent higher frailty around the T3 → T4 interval was one of the more stable findings, remaining evident in the main RI-CLPM and persisting across sensitivity analyses more consistently than most other severity-related paths. Second, the association between higher frailty and subsequent higher symptom interference in the earliest interval, T0 → T1, was also relatively recurrent across specifications, even when the reverse-direction interference-to-frailty path was not stable. These two patterns therefore stand out as the most reproducible signals in the present analyses.

In relation to the existing literature, these findings both align with and refine prior work. They align with studies showing close longitudinal links between frailty and symptom burden in older adults, but they refine that literature by suggesting that the relationship may not be globally reciprocal or temporally uniform. Instead, different symptom dimensions may align with frailty at different phases of recovery, and some apparent longitudinal reciprocity may weaken after stable between-person differences are separated. Thus, the present study contributes not a single directional claim but a more differentiated account: frailty and symptom burden were closely linked at the person level, while within-person longitudinal associations were selective, subscale-specific, and sensitive to model specification.

### 4.9. Limitations

This study has several limitations.

First, this single-center study was conducted at Shanghai Tenth People’s Hospital, an urban tertiary institution equipped with standardized surgical services, serving predominantly Shanghai residents (74%) with relatively high education levels (42.2% with high school or above). These characteristics may limit generalizability to: (1) rural or resource-limited settings; (2) patients with lower socioeconomic status or health literacy; (3) non-Chinese populations with different surgical care pathways; and (4) colorectal cancer populations with advanced stage or poor performance status (our cohort had only 5% postoperative complications), which may experience different recovery trajectories.

Second, both frailty and symptom burden relied on self-reported instruments. Although the distinction between MDASI symptom severity and symptom interference allowed a more differentiated analysis of symptom burden, both domains remained based on patient-reported measures. Incorporation of objective biomarkers and performance-based indicators, such as inflammatory markers (e.g., IL-6, TNF-α, CRP), sarcopenia indicators via DEXA, grip strength, gait speed, and accelerometer-derived physical activity, would strengthen future longitudinal inference and provide complementary information beyond self-reported instruments.

Third, measurement intervals were unequal (from 1 week to 6 months between assessments), which may have obscured more rapid symptom–frailty fluctuations occurring on shorter timescales. This limitation in temporal resolution may have reduced the ability to detect short-term within-person dynamic associations, particularly during the early postoperative phase when recovery-related changes are often most rapid.

Fourth, residual time-varying confounding represents a significant threat to internal validity. Although we adjusted for two time-varying nutritional indicators (serum albumin and BMI) in sensitivity analyses and core results remained stable, a wide range of postoperative time-varying exposures may jointly shape symptom burden and frailty trajectories, including: (1) timing and cycles of adjuvant chemotherapy, (2) analgesic regimen adjustments and opioid dose escalation, (3) frequency of postoperative rehabilitation participation, (4) postoperative infectious complications such as anastomotic leakage, (5) functional impairment associated with stoma care, and (6) psychological distress including persistent fear of cancer recurrence. Since these clinically relevant time-varying covariates were not repeatedly measured across all follow-up waves, we cannot fully exclude the possibility that unmeasured confounding biases the within-person symptom-to-frailty cross-lagged associations.

Fifth, although this study separated MDASI symptom severity and symptom interference, it still relied on subscale-level summary scores and did not examine distinct symptom clusters or individual symptoms separately. Different symptom domains may show different longitudinal association patterns with frailty. For example, pain and fatigue may have different temporal relationships with frailty than gastrointestinal symptoms or emotional distress. Similarly, the GFI captures multidimensional frailty but does not distinguish in detail among physical, cognitive, social, and psychological frailty subdomains, which may show different associations with specific symptom dimensions. Thus, the current analyses may have obscured more granular heterogeneity within both symptom burden and frailty.

Sixth, multiple interval-specific autoregressive and cross-lagged parameters were tested across the two RI-CLPMs and across sensitivity analyses, but no formal multiplicity correction was applied. As a result, some statistically significant findings, particularly isolated or borderline interval-specific associations, may represent chance findings. Accordingly, these path-specific results should be interpreted as exploratory pattern-detection rather than as definitive evidence for a stable longitudinal association at every interval, and replication in independent datasets would be important.

Seventh, selection bias may have influenced the results. Patients with severe complications or poor functional status may have preferentially declined study participation or experienced early dropout (15.3% monotone dropout). Although baseline comparisons and FIML estimation were performed, participants with complete data differed in BMI, suggesting that baseline heterogeneity may not have been fully captured. This may have biased some estimates toward the null if participants with stronger frailty–symptom associations were more likely to drop out or remain underrepresented.

### 4.10. Future Directions

Future research directions can be expanded along mechanistic, methodological, and translational lines.

First, greater mechanistic specificity is needed. Analyses of distinct symptom clusters, such as pain–fatigue–sleep disturbance clusters and gastrointestinal–emotional clusters, as well as analyses of individual symptoms, may reveal more specific longitudinal association patterns than subscale-level symptom summaries alone. Likewise, separate examination of frailty subdomains, including physical, cognitive, psychological, and social dimensions, may help clarify which aspects of frailty are most closely aligned with different types of symptom burden. In addition, incorporation of objective measures, including inflammatory markers (IL-6, TNF-α, and CRP), sarcopenia indicators via DEXA, grip strength, gait speed, and accelerometer-derived physical activity, would strengthen future longitudinal studies by complementing self-reported assessments.

Second, methodological refinement is essential. Multicenter external validation is needed to establish generalizability beyond the single-center urban Chinese cohort studied here. Denser assessment schedules within the critical postoperative window, particularly between 1 and 6 months, would better resolve the temporal ordering of symptom fluctuations and frailty transitions. Future studies should also prospectively and repeatedly document clinically relevant time-varying exposures at each assessment wave, including chemotherapy exposure status and cycle number; medication adjustments, especially analgesic regimens; rehabilitation participation frequency and intensity; objective physical activity; infectious complications; unplanned healthcare utilization; stoma-specific difficulties when applicable; and psychological distress, including persistent fear of cancer recurrence. More systematic measurement of these factors would allow a more rigorous evaluation of whether observed symptom–frailty associations persist after more comprehensive control of time-varying confounding. In addition, future studies should prespecify how multiplicity will be handled when large numbers of interval-specific paths are examined, for example, through false discovery rate control, family-wise error procedures, or emphasis on replication of prespecified key paths rather than isolated nominally significant findings. Strategies to minimize attrition among patients with severe complications or poor functional status, such as flexible assessment modalities, closer follow-up schedules, home-based or telephone-based data collection, and inverse probability weighting or related approaches, should also be incorporated to mitigate selection bias.

Third, translational research should move beyond observational modeling to intervention-based designs. Ultimately, randomized controlled trials targeting structured, protocolized symptom management during the postoperative months, especially during the intervals in which the present study suggested more consistent symptom–frailty associations, would be needed to determine whether reducing symptom burden is accompanied by more favorable frailty trajectories. Such trials should prespecify frailty subdomain endpoints and incorporate objective indicators, such as grip strength, gait speed, or inflammatory markers, alongside patient-reported outcomes in order to capture multidimensional changes. If benefit is shown, integrating structured symptom monitoring and management into existing Enhanced Recovery After Surgery pathways may offer a practical route for improving longer-term functional outcomes in older colorectal cancer survivors.

## 5. Conclusions

In this longitudinal study of older adults undergoing colorectal cancer surgery, frailty was consistently associated with higher symptom burden at the between-person level, whereas within-person cross-lagged associations were interval-specific and differed between MDASI symptom severity and symptom interference. The relatively more stable findings were a positive association between higher symptom severity and subsequent higher frailty around the middle follow-up interval and a positive association between higher frailty and subsequent higher symptom interference in the earliest postoperative interval. Sensitivity analyses showed that several path estimates varied across model specifications, nutritional adjustment, and assumptions about missingness. Overall, the findings support a heterogeneous, phase-specific, and model-dependent relationship between frailty and postoperative symptom burden rather than a single uniform longitudinal pattern.

## Figures and Tables

**Table 1:** Baseline characteristics of participants (N = 242).

Characteristic	*N* = 242 ^1^
Sex, n (%)	
Male	138 (57.0%)
Female	104 (43.0%)
Age, years	69.87 ± 7.61
Residence, n (%)	
Shanghai	179 (74.0%)
Other provinces	63 (26.0%)
Education level, n (%)	
Junior high school or below	140 (57.8%)
Senior high school	72 (29.8%)
College/associate degree or above	30 (12.4%)
Marital status, n (%)	
Married	193 (79.8%)
Other	49 (20.2%)
Diagnosis, n (%)	
Colon/sigmoid cancer	160 (66.1%)
Rectal cancer	82 (33.9%)
BMI, kg/m^2^	23.43 ± 3.12
NRS 2002 score	1.54 ± 0.95
Nutritional risk (NRS 2002), n (%)	
Low risk (NRS < 3)	215 (88.8%)
At risk (NRS ≥ 3)	27 (11.2%)
Stoma, n (%)	
Yes	40 (16.5%)
No	202 (83.5%)
Postoperative complications, n (%)	
Yes	12 (5.0%)
No	230 (95.0%)
Hemoglobin, g/L	117.42 ± 24.09
Albumin, g/L	38.45 ± 4.32
Total protein, g/L	65.56 ± 7.30

^1^ n (%); Mean ± SD.

**Table 2:** Missing-data pattern across six assessment time points (*N* = 242).

Time Point	Interval	GFI			MDASI		
		n Complete	n Missing	% Missing	n Complete	n Missing	% Missing
T0	Baseline (pre-surgery)	242	0	0.0%	242	0	0.0%
T1	1 week post-surgery	242	0	0.0%	242	0	0.0%
T2	1 month post-surgery	233	9	3.7%	233	9	3.7%
T3	3 months post-surgery	223	19	7.9%	223	19	7.9%
T4	6 months post-surgery	211	31	12.8%	210	32	13.2%
T5	12 months post-surgery	205	37	15.3%	204	38	15.7%

Note. GFI = Groningen Frailty Indicator; MDASI = M.D. Anderson Symptom Inventory. Missing data were handled using full-information maximum likelihood (FIML) estimation. A total of 9 distinct missing-data patterns were identified across the 12 observed variables. Attrition followed a monotone pattern with missingness concentrated at later time points, consistent with typical postoperative longitudinal follow-up studies.

**Table 3:** Detailed missing data patterns across six measurement waves for GFI and MDASI.

Pattern ID	T0	T1	T2	T3	T4	T5	N	Percentage (%)	Description
1	O	O	O	O	O	O	202	83.5%	Completely observed data
2	O	O	O	O	O	M	6	2.5%	Monotone dropout from T5 onwards
3	O	O	O	O	M	M	12	5.0%	Monotone dropout from T4 onwards
4	O	O	O	M	M	M	10	4.1%	Monotone dropout from T3 onwards
5	O	O	M	M	M	M	9	3.7%	Monotone dropout from T2 onwards
6	O	O	O	O	M	O	1	0.4%	Intermittent missingness at T4
7	O	O	O	M	O	O	1	0.4%	Intermittent missingness at T3
8	O	O	O	O	O (GFI)/M (MDASI)	M	1	0.4%	MDASI missing at T4, both missing at T5
Total							242	100%	

Note: O indicates observed data for both GFI and MDASI at the respective time point. M indicates missing data for both GFI and MDASI at the respective time point. For Pattern 8, GFI data were observed while MDASI data were missing at T4, and both GFI and MDASI data were missing at T5. GFI = Groningen Frailty Indicator; MDASI = M.D. Anderson Symptom Inventory.

**Table 4:** Descriptive statistics for frailty (GFI) and symptom burden (MDASI) across six time points.

Time Point	Frailty		Symptom Burden			
	GFI (Mean ± SD)	*N*	MDASI Severity (Mean ± SD)	*N*	MDASI Interference (Mean ± SD)	*N*
T0	2.83 ± 2.48	242	1.12 ± 0.93	242	2.00 ± 1.65	242
T1	4.60 ± 2.20	242	1.42 ± 0.99	242	3.20 ± 1.78	242
T2	3.48 ± 2.49	233	1.40 ± 1.15	233	2.35 ± 1.89	233
T3	2.98 ± 2.17	223	1.44 ± 1.18	223	1.82 ± 1.60	223
T4	3.41 ± 2.83	211	1.41 ± 1.49	210	1.76 ± 2.04	210
T5	2.53 ± 2.72	205	0.89 ± 1.06	204	1.15 ± 1.82	204

Note: T0, baseline (preoperative); T1, 1 week postoperatively; T2, 1 month postoperatively; T3, 3 months postoperatively; T4, 6 months postoperatively; T5, 12 months postoperatively. Values are reported as M ± SD. *N* denotes participant count at each time point. Both constructs showed peak values at T1 and subsequently declined, with minimum values observed at T5.

**Table 5:** RI-CLPM results for frailty and MDASI symptom severity.

Path	Interval	B	SE	*β*	95% CI
GFI → MDASI					
c1: T0 → T1	Baseline → 1 week	−0.009	0.043	−0.020	[−0.203, 0.162]
c2: T1 → T2	1 week → 1 month	0.012	0.046	0.023	[−0.148, 0.195]
c3: T2 → T3	1 month → 3 months	0.022	0.046	0.044	[−0.135, 0.223]
c4: T3 → T4	3 months → 6 months	−0.199	0.080	−0.231	[−0.414, −0.049]
c5: T4 → T5	6 months → 12 months	−0.041	0.045	−0.095	[−0.301, 0.111]
MDASI → GFI					
d1: T0 → T1	Baseline → 1 week	0.309	0.174	0.165	[−0.018, 0.348]
d2: T1 → T2	1 week → 1 month	−0.057	0.163	−0.030	[−0.202, 0.142]
d3: T2 → T3	1 month → 3 months	0.308	0.142	0.195	[0.018, 0.373]
d4: T3 → T4	3 months → 6 months	1.061	0.213	0.439	[0.265, 0.613]
d5: T4 → T5	6 months → 12 months	−0.211	0.176	−0.124	[−0.329, 0.080]
GFI Autoregressive					
a1: T0 → T1	Baseline → 1 week	0.037	0.077	0.045	[−0.138, 0.228]
a2: T1 → T2	1 week → 1 month	−0.048	0.091	−0.046	[−0.218, 0.126]
a3: T2 → T3	1 month → 3 months	0.075	0.073	0.092	[−0.086, 0.270]
a4: T3 → T4	3 months → 6 months	−0.532	0.135	−0.346	[−0.520, −0.172]
a5: T4 → T5	6 months → 12 months	0.166	0.097	0.177	[−0.028, 0.381]
MDASI Autoregressive					
b1: T0 → T1	Baseline → 1 week	0.239	0.097	0.227	[0.045, 0.409]
b2: T1 → T2	1 week → 1 month	−0.103	0.083	−0.108	[−0.279, 0.064]
b3: T2 → T3	1 month → 3 months	0.200	0.090	0.203	[0.023, 0.382]
b4: T3 → T4	3 months → 6 months	0.285	0.125	0.211	[0.028, 0.394]
b5: T4 → T5	6 months → 12 months	−0.000	0.082	−0.000	[−0.206, 0.206]

Note: GFI = Groningen Frailty Indicator; MDASI = M. D. Anderson Symptom Inventory; B = unstandardized coefficient; SE = standard error; *β* = standardized coefficient. The 95% confidence intervals reported for within-person paths correspond to *β*.

**Table 6:** RI-CLPM results for frailty and MDASI symptom interference.

Path	Interval	B	SE	*β*	95% CI
GFI → MDASI					
c1: T0 → T1	Baseline → 1 week	0.197	0.063	0.284	[0.105, 0.462]
c2: T1 → T2	1 week → 1 month	0.072	0.074	0.091	[−0.093, 0.274]
c3: T2 → T3	1 month → 3 months	0.121	0.062	0.177	[−0.003, 0.356]
c4: T3 → T4	3 months → 6 months	0.009	0.111	0.008	[−0.176, 0.191]
c5: T4 → T5	6 months → 12 months	−0.008	0.075	−0.012	[−0.220, 0.196]
MDASI → GFI					
d1: T0 → T1	Baseline → 1 week	−0.255	0.102	−0.227	[−0.405, −0.049]
d2: T1 → T2	1 week → 1 month	0.127	0.117	0.101	[−0.083, 0.286]
d3: T2 → T3	1 month → 3 months	0.064	0.098	0.059	[−0.120, 0.238]
d4: T3 → T4	3 months → 6 months	0.140	0.165	0.079	[−0.104, 0.261]
d5: T4 → T5	6 months → 12 months	−0.112	0.129	−0.091	[−0.300, 0.117]
GFI Autoregressive					
a1: T0 → T1	Baseline → 1 week	0.272	0.075	0.328	[0.150, 0.506]
a2: T1 → T2	1 week → 1 month	−0.143	0.097	−0.138	[−0.322, 0.046]
a3: T2 → T3	1 month → 3 months	0.151	0.074	0.186	[0.007, 0.365]
a4: T3 → T4	3 months → 6 months	−0.161	0.142	−0.105	[−0.287, 0.078]
a5: T4 → T5	6 months → 12 months	0.144	0.099	0.153	[−0.055, 0.361]
MDASI Autoregressive					
b1: T0 → T1	Baseline → 1 week	−0.123	0.085	−0.131	[−0.310, 0.048]
b2: T1 → T2	1 week → 1 month	−0.041	0.089	−0.043	[−0.227, 0.141]
b3: T2 → T3	1 month → 3 months	0.051	0.083	0.056	[−0.123, 0.236]
b4: T3 → T4	3 months → 6 months	−0.158	0.129	−0.114	[−0.297, 0.069]
b5: T4 → T5	6 months → 12 months	0.115	0.097	0.125	[−0.083, 0.333]

Note. GFI = Groningen Frailty Indicator; MDASI = M. D. Anderson Symptom Inventory; B = unstandardized coefficient; SE = standard error; *β* = standardized coefficient. The 95% confidence intervals reported for within-person paths correspond to *β*.

## Data Availability

The data presented in this study are available upon request from the corresponding author. The data are not publicly available due to privacy and ethical restrictions regarding patient confidentiality.

## References

[1] Sung H., Filho A.M., Laversanne M., Ferlay J., Siegel R.L., Soerjomataram I., Jemal A., Bray F. (2026). Global Cancer Statistics 2024: GLOBOCAN Estimates of Incidence and Mortality Worldwide for 34 Cancers in 186 Countries. CA A Cancer J. Clin..

[2] Morris V.K., Kennedy E.B., Baxter N.N., Benson A.B., Cercek A., Cho M., Ciombor K.K., Cremolini C., Davis A., Deming D.A. (2023). Treatment of Metastatic Colorectal Cancer: ASCO Guideline. J. Clin. Oncol..

[3] Tsai H.-F., Liu C.-Y., Yang S.-H., Chiou A.-F. (2022). Factors Related to Frailty in Older Cancer Patients Undergoing Colorectal Surgery: A Longitudinal Study. Cancer Nurs..

[4] Normann M., Ekerstad N., Angenete E., Prytz M. (2025). Frailty Is an Independent Marker of Post-Operative Mortality Following Colorectal Cancer Resection Surgery in Older Adults. J. Surg. Oncol..

[5] Han J., Zhang Q., Lan J., Yu F., Liu J. (2024). Frailty Worsens Long-Term Survival in Patients with Colorectal Cancer: A Systematic Review and Meta-Analysis. Front. Oncol..

[6] Wu I.H.C., Balachandran D.D., Faiz S.A., Bashoura L., Escalante C.P., Manzullo E.F. (2022). Characteristics of Cancer-Related Fatigue and Concomitant Sleep Disturbance in Cancer Patients. J. Pain Symptom Manag..

[7] Calderón C., Jiménez-Fonseca P., Hernández R., Mar Muñoz M.D., Mut M., Mangas-Izquierdo M., Vicente M.Á., Ramchandani A., Carmona-Bayonas A. (2019). Quality of Life, Coping, and Psychological and Physical Symptoms after Surgery for Non-Metastatic Digestive Tract Cancer. Surg. Oncol..

[8] Liao Y., Liu X., Wu X., Li C., Li Y. (2024). Social Isolation Profiles and Conditional Process Analysis among Postoperative Enterostomy Patients with Colorectal Cancer. BMC Psychol..

[9] Van Rooijen S., Carli F., Dalton S., Thomas G., Bojesen R., Le Guen M., Barizien N., Awasthi R., Minnella E., Beijer S. (2019). Multimodal Prehabilitation in Colorectal Cancer Patients to Improve Functional Capacity and Reduce Postoperative Complications: The First International Randomized Controlled Trial for Multimodal Prehabilitation. BMC Cancer.

[10] Zhang J., He Q., Wang M., Lv X., Mao D., Li J., Zhu D., Huang L. (2025). Core Symptoms in Patients with Colorectal Cancer Receiving Chemotherapy: A Network Analysis. Support. Care Cancer.

[11] Shang B., Bian Z., Luo C., Lv F., Wu J., Lv S., Wei Q. (2024). Exploring the Dynamics of Perioperative Symptom Networks in Colorectal Cancer Patients: A Cross-Lagged Panel Network Analysis. Support. Care Cancer.

[12] Han C.J., Yang G.S., Syrjala K. (2020). Symptom Experiences in Colorectal Cancer Survivors After Cancer Treatments: A Systematic Review and Meta-Analysis. Cancer Nurs..

[13] Zhong M., Qi B., Sun Y., Shi Y., Cao Q. (2026). Symptom Network in Patients with Colorectal Cancer during the Perioperative Period. Support. Care Cancer.

[14] Li Y., Gan W., Mao Q., Wu H., Cao T., Wu H., Mao X. (2025). Trajectories of Symptom Clusters and Their Predictive Factors in Patients with Colorectal Cancer 3 Months After Surgery: A Longitudinal Study. Cancer Med..

[15] Maeda H., Takahashi M., Seo S., Hanazaki K. (2023). Frailty and Colorectal Surgery: Review and Concept of Cancer Frailty. J. Clin. Med..

[16] Chen K., Yang D., Li F., Gao L., Tian Y., Xu B., Xu X., Xu Q., Cao J. (2023). Changes in the Symptom Clusters of Elderly Patients with Lung Cancer over the Course of Postoperative Rehabilitation and Their Correlation with Frailty and Quality of Life: A Longitudinal Study. Eur. J. Oncol. Nurs..

[17] Ho M.-H., Cheung D.S.T., Chan W.L., Lin C.-C. (2023). Cognitive Frailty in Older Cancer Survivors and Its Association with Health-Related Quality of Life. Eur. J. Oncol. Nurs..

[18] Hallet J., Tillman B., Zuckerman J., Guttman M.P., Chesney T., Mahar A.L., Chan W.C., Coburn N., Haas B. (2022). Association Between Frailty and Time Alive and At Home After Cancer Surgery Among Older Adults: A Population-Based Analysis. J. Natl. Compr. Cancer Netw..

[19] Zhou W., Cho Y., Shang S., Li Y., Seo J., Pu J., Song R. (2026). Frailty Transitions and Associated Factors in Older Cancer Survivors in the USA: Using a Multi-State Modeling. J. Cancer Surviv..

[20] Zheng B.Q., Valente M.J. (2023). Evaluation of Goodness-of-Fit Tests in Random Intercept Cross-Lagged Panel Model: Implications for Small Samples. Struct. Equ. Model. A Multidiscip. J..

[21] Hoffman L., Hall G.J. (2024). Considering Between- and within-Person Relations in Auto-Regressive Cross-Lagged Panel Models for Developmental Data. J. Sch. Psychol..

[22] Wolf E.J., Harrington K.M., Clark S.L., Miller M.W. (2013). Sample Size Requirements for Structural Equation Models: An Evaluation of Power, Bias, and Solution Propriety. Educ. Psychol. Meas..

[23] Christopher Westland J. (2010). Lower Bounds on Sample Size in Structural Equation Modeling. Electron. Commer. Res. Appl..

[24] Apriadi D., Pimolkatekul S., Susanti E., Chiu H.-Y., Huang H.-C. (2025). Diagnostic Accuracy of Self-Reported Tools for Frailty Assessment in Older Adults with Cancer: A Diagnostic Meta-Analysis. Int. J. Older People Nurs..

[25] Cleeland C.S., Mendoza T.R., Wang X.S., Chou C., Harle M.T., Morrissey M., Engstrom M.C. (2000). Assessing Symptom Distress in Cancer Patients: The M. D. Anderson Symptom Inventory. Cancer.

[26] Wang X.S., Wang Y., Guo H., Mendoza T.R., Hao X., Cleeland C.S. (2004). Chinese Version of the M. D. Anderson Symptom Inventory: Validation and Application of Symptom Measurement in Cancer Patients. Cancer.

[27] Sukpan C., Kuiper R.M. (2026). Selecting the Correct RI-CLPM Using Chi-Square-Type Tests and AIC-Type Criteria. Struct. Equ. Model. A Multidiscip. J..

[28] Mund M., Johnson M.D., Nestler S. (2021). Changes in Size and Interpretation of Parameter Estimates in Within-Person Models in the Presence of Time-Invariant and Time-Varying Covariates. Front. Psychol..

[29] Zulkifli R., Aimran N., Deni S.M., Badarisam F.N. (2022). A Comparative Study on the Performance of Maximum Likelihood, Generalized Least Square, Scale-Free Least Square, Partial Least Square and Consistent Partial Least Square Estimators in Structural Equation Modeling. Int. J. Data Netw. Sci..

